# The Role of Vascular Cells in Pancreatic Beta-Cell Function

**DOI:** 10.3389/fendo.2021.667170

**Published:** 2021-04-26

**Authors:** Guzel Burganova, Claire Bridges, Peter Thorn, Limor Landsman

**Affiliations:** ^1^ Department of Cell and Development Biology, Sackler Faculty of Medicine, Tel Aviv University, Tel Aviv, Israel; ^2^ Charles Perkins Centre, Faculty of Medicine and Health, University of Sydney, Camperdown, NSW, Australia

**Keywords:** islet vasculature, endothelial cells, pericytes, beta-cells, Islets of Langerhans, basement membrane

## Abstract

Insulin-producing β-cells constitute the majority of the cells in the pancreatic islets. Dysfunction of these cells is a key factor in the loss of glucose regulation that characterizes type 2 diabetes. The regulation of many of the functions of β-cells relies on their close interaction with the intra-islet microvasculature, comprised of endothelial cells and pericytes. In addition to providing islet blood supply, cells of the islet vasculature directly regulate β-cell activity through the secretion of growth factors and other molecules. These factors come from capillary mural pericytes and endothelial cells, and have been shown to promote insulin gene expression, insulin secretion, and β-cell proliferation. This review focuses on the intimate crosstalk of the vascular cells and β-cells and its role in glucose homeostasis and diabetes.

## Introduction

Pancreatic β-cells reside in a complex microenvironment, where they interact with other endocrine cells, as well as vascular endothelial cells and pericytes, immune cells and neurons (1–4). This review focuses on the crosstalk of β-cells with vascular cells and their role in glucose homeostasis and diabetes.

Islets, comprised of highly vascularised clusters of endocrine cells, are the functional units within the pancreas that control blood sugar levels. A dense capillary network surrounds and penetrates each pancreatic islet to enable glucose sensing and insulin secretion into peripheral circulation (5–8). While representing only 1–2% of the pancreatic mass, islets receive up to 20% of the direct arterial blood flow to the pancreas (9). Structural and phenotypical analysis of the pancreatic vasculature demonstrate a dense network of thick, highly-branched capillaries within islets (1, 10) - due to this high level of vascularisation, almost all β-cells come into contact with a capillary (11, 12). Pancreatic capillary structure consists of a highly-fenestrated, luminal layer of endothelial cells (13) surrounded by pericytes, which are abluminal mural cells embedded within the microvessel basement membrane (14). While there is a large body of data surrounding the roles of endothelial cells in the function of capillary beds (15–20), the specific roles of pericytes are complex and not fully understood. However, recent studies showed that pericytes play a vital role in regulating β-cell function and mass (21–24). Both types of islet vascular cells are known to promote insulin production and secretion, as well as β-cell proliferation, survival, and maturation, by secreting a variety of growth factors, components of the extracellular matrix (ECM), and other molecules (5, 22, 23, 25–27).

β-Cells do not appear to directly contact vascular cells, instead, a double-layered basement membrane comprised of extracellular matrix (ECM) glycoproteins surrounds islet capillaries in both humans (28) and mice (29), lying between the vascular and β-cells. It is apparent that β-cells specifically respond to regional contact with the capillaries. Each β-cell is structurally polarised with a basal domain at the point of capillary contact and an apical domain positioned away from the capillaries (12, 30). Synaptic scaffold proteins are enriched in this basal domain and insulin granule fusion is selectively targeted to this region (12, 31).

Vascular cells in the islets are involved in tissue inflammation and immunoregulation. Endothelial cells recruit macrophages, which in turn induce β-cell proliferation and regeneration (5–7). Although the immunoregulatory properties of pericytes in other tissues, such as the brain (32, 33) and kidney (34), are well-established, whether pancreatic pericytes have similar capabilities in the islet have yet to be reported.

Accumulating evidence is showing that the interactions between vascular cells and β-cells are essential for correct islet development and become key factors in the regulation of adult islet function.

## Role of Vascular Cells in Islet Embryonic Development

The development of many tissues, including the pancreas, depends on the interactions of various cell types. Pancreatic endocrine and exocrine cells originate from the foregut endoderm and acquire their differentiated fate in a sequential process (21–24, 35). Cells of the embryonic pancreatic microenvironment, including endothelial and mesenchymal cells, have been shown to regulate this process (24, 36).

The embryonic pancreatic mesenchyme regulates pancreas organogenesis, primarily through promoting appropriate survival and proliferation of endoderm-derived cells (37). In the final stages of embryonic development, pericytes originate from the pancreatic mesenchyme (24, 38), which stimulates the replication of differentiated β-cells (37, 38). β-Cells continue to proliferate during the neonatal stage (39), and Diphtheria Toxin-mediated depletion of neonatal pericytes results in a reduced rate of β-cells replication demonstrating the influence of pericytes on β-cell expansion in both the embryonic and neonatal pancreata (22).

Endothelial cells also play a key role in islet development. The reciprocal influences of β-cells and islet endothelial cells affect development of both the vasculature (40) and β-cells (41, 42). Evidence indicates that β-cells are unlikely to synthesize their own ECM components, and that developmental expression of angiogenic protein VEGFA from β-cells is vital to encourage the islet vascularisation required for basement membrane formation in developing islets (25, 43).

Given that the islets comprise an endocrine organ and are therefore dependent on close coupling with the whole-body blood circulation, it is not surprising that vascular cells such as pericytes and endothelial cells are important for islet development. The uncovering of the roles and mechanisms of these vascular cells is interesting and potentially important for cell-based treatments for diabetes.

## Vascular Regulation of Adult Islet Function

Exactly how pericytes and endothelial cells influence β-cell function in an adult islet is a developing area of study (44) and can, in principle, occur through a variety of effects including; capillary behaviour, secreted factors, direct contact, or ECM-driven interactions.

### The Role of Vascular Cells in Capillary Behaviour

Islet blood flow is obviously important for endocrine function and allows the rapid sensing of fluctuations in blood glucose and outflow of secreted hormones. It is controlled by various nutrients and growth factors (1) and, in turn, impacts on β-cell insulin secretory activity (4, 45).

Approximately 40% of islet microvasculature is covered by pericytes (21) which adjust the vascular diameter and capillary blood flow by vasoconstriction and vasodilation (46). Research in this area has identified several molecules responsible for the regulation of pericyte contractile tone. For example, pericytes in the brain have been shown to express receptors for vasoactive molecules (47). Endothelial cells are known to secrete vasoactive factors, including vasodilators nitric oxide and prostacyclin as well as vasoconstrictors thromboxane and endothelin-1 (48). In the pancreas, adenosine released during ATP breakdown increases islet blood flow (49) and relaxes pericytes to dilate islet capillaries (21). In contrast, the sympathetic neurotransmitter noradrenaline induces contraction of islet capillaries and reduces blood flow (21). The cellular contacts and paracrine signalling between endothelial cells and pericytes that regulate vascular tone likely influence blood flow effects on β-cell endocrine function.

### The Role of Secreted Factors From Vascular Cells

There is now extensive evidence that pericytes directly support β-cell function and glucose homeostasis independent of blood flow (6, 22, 23, 26, 27). *In vivo* depletion of pericytes in the pancreas using the Diphtheria Toxin Receptor system allows study of the role in β-cell function and proliferation (22, 27). This depletion of pancreatic pericytes leads to glucose intolerance due to reduced islet insulin content and secretion, as well as diminished expression of cellular components required for β-cell functionality. Importantly, reduced levels of *MafA* and *Pdx1*, transcription factors essential for β-cell maturity, indicate β-cell de-differentiation occurs in the absence of pericytes (27). Pancreatic pericytes are further shown to secrete factors that regulate glucose-stimulated insulin secretion (GSIS). Pancreatic mural cells, i.e., pericytes and vascular smooth muscle cells, produce nerve growth factor (NGF) upon glucose stimulation (23). β-Cells express the NGF receptor tropomyosin receptor kinase A (TrkA), and the activation of this receptor promotes insulin exocytosis *via* glucose-induced β-cell actin remodelling (23). In humans, altered circulating NGF levels have been noted in type 2 diabetes and mutations in the TrkA gene cause decreased GSIS (50, 51). Pericytes further produce Bone morphogenetic protein 4 (BMP4), through which they potentially directly regulate β-cell function (26). While the activity of the BMP4 receptor BMPR1A is essential for proper β-cell gene expression and function (52), the involvement of pericytic BMP4 in this process was yet to be reported. The evidence of glucose-stimulated paracrine signaling between pericytes and β-cells highlights the importance of pericytes in glucose homeostasis and GSIS under physiological conditions.

Among the many factors secreted by intra-islet endothelial cells, connective tissue growth factor (CTGF) and thrombospondin (TSP)-1 have known effects on β-cells (53). CTGF, a matricellular protein active throughout the body (54), drives β-cell expansion during embryogenesis in an autocrine manner (55), thought to occur due to multiple development-related transcription factor binding sites located on the CTGF gene although a mechanism has not yet been clearly defined (56). In the adult pancreas, CTGF is expressed mostly by islet endothelial cells (57). Islets that underwent partial destruction of β-cell content were treated with CTGF, leading to a 50% mass recovery attributed to the proliferative effects of the growth hormone (58). Production of TSP-1, an anti-angiogenic protein secreted by intra-islet endothelial cells, is upregulated by elevated blood glucose levels in humans (59). TSP-1-deficiency, however, leads to pancreatic hyperplasia, glucose intolerance, and impaired GSIS (60) despite knockdown-related improvements in transplanted islet revascularization (61). Rescue of TSP-1-deficient murine islets through treatment with transforming growth factor (TGF) β-1 activation inhibits the decreased glucose tolerance (60), providing insight into potential mechanisms. However, long-term deficiency of TSP-1 results in persistent dysfunction of glucose tolerance, even in the face of compensatory normalisation of β-cell mass (62). Additional molecules produced in non-pancreatic endothelial cells, such as hepatocyte growth factor (HGF), influence β-cell function as well *via* exocrine signaling (63).

### Direct Contacts Between Vascular Cells and β-Cells

Due to the structure of the double-layered basement membrane (28, 64), β-cells are unlikely to make direct contact with intra-islet vascular endothelial cells or pericytes. However, there are candidate proteins that might indicate direct links are possible. For example, the pre- and post-synaptic proteins neurexin and neuroligin are expressed by vascular mural and endothelial cells (65), and have additionally been identified in β-cells (66). In neurons, these binding partners directly contact each other and mediate a plethora of biological functions (67) including synaptic organisation. Over-expression of post-synaptic receptor neuroligin-2 expression in β-cells increases GSIS (68) and promotes insulin granule docking (69). The neurexin-neuroligin interactions therefore appear to be involved in regulating β-cell function. These interactions may arise through β-cell-to-β-cell contacts, but it remains an intriguing possibility that they result from interactions between the β-cells and the vascular cells. Connexins 36 and 43, which form gap junctions between cells, are similarly expressed by both endothelial and β-cells, however there is currently no evidence demonstrating direct contacts between the two cell types (44).

### Interactions of β-Cells With the Basement Membrane

Various proteins of the vascular basement membrane are implicated in the regulation of β-cell function, proliferation, and expansion (22, 25, 64, 70). The basement membrane is comprised of glycoproteins including laminins, fibronectin, nidogens, and collagens (29, 71). The basement membrane surrounds the intra-islet capillaries and the islet capsule but is not present between endocrine cells (29); therefore, β-cells contact the basement membrane only in the regions which they contact the vasculature (25, 30). Evidence demonstrates that these contacts, mediated through integrin activation (31), assist in driving β-cell polarity and the targeting of insulin secretion (12, 31) in addition to modulating insulin gene expression (25), β-cell proliferation and survival (72), and GSIS functionality (73, 74).

Both endothelial and pericytes secrete ECM components that make up the islet basement membrane. Endothelial cells are responsible for the synthesis and maintenance of the ECM/basement membrane, specifically producing laminin and collagen IV (25, 75, 76). Pancreatic pericytes also produce an array of basement membrane components, including collagen IV, laminins, proteoglycans, and nidogen (77). In particular, pancreatic pericytes and endothelial cells both produce laminin α4, which promotes the expression levels of the β-cell genes *Ins1*, *MafA*, and *Glut2*, as well as GSIS (77).

*In vitro* research surrounding the function of pancreatic islets is largely performed with cells derived from isolated islets, obtained through enzymatic destruction of the ECM structure (78, 79). Although islets retain some endothelial cell expression immediately post-isolation, the endothelial cells are rapidly diminished during culture with islets losing approximately 85% of endothelial cells within two days of culture (80). This loss of vasculature negatively impacts the endocrine function of isolated islets (25). Various lines of evidence show that attempts to preserve, restore or replace the vascular cells is beneficial to β-cells. Endothelial cell-conditioned medium in culture of dispersed β-cells improves GSIS with a laminin-dependent mechanism (81). Similarly, exposure to pericyte-conditioned medium stimulates proliferation in cultured β-cells in an integrin-dependent manner (10, 22). In islet transplantation, supplementing islets with endothelial cells improves revascularisation and functional outcomes compared to islets alone (82–84).

Additionally, simple incorporation of basement membrane proteins into cell cultures has repeatedly been shown to benefit cultured β-cell function and survival and is furthermore a useful approach to gain a mechanistic understanding of the processes involved. Introduction of laminins α4 and α5 to β-cells cultured *in vitro* on glass increases insulin gene expression and enhances GSIS, effects that are inhibited by the blockade of the integrin β1 receptor (25). β1 integrin has been demonstrated to regulate GSIS (85) as well as β-cell expansion (70), furthering the evidence that β1 integrins play a key role in ECM influences on β-cell endocrine function.

In addition to affecting β-cell function, ECM contacts between islet vasculature and β-cells contribute to β-cell polarity (12, 30) and likely orientate the site of targeted insulin secretion to the capillaries (12, 31). Targeting of insulin granule fusion appears to be driven by the localisation of pre-synaptic scaffold proteins, including liprin, RIM2, piccolo, and ELKS, at the contact point of β-cells and the islet vasculature (12, 86) and has been shown to depend on localised β1 integrin activation (31).

Contact between β-cells and ECM triggers focal adhesion formation downstream of β1 integrin activation, shown *via* immunostaining to occur exclusively at the interface between islet blood vessels and β-cells (31). As insulin granule fusion is biased towards this interface (12, 31), current evidence indicates this targeting of secretion requires the focal adhesion activation and the specific involvement of focal adhesion kinase (FAK). Evidence suggests FAK as a vital signaling mediator for β-cell endocrine function, as pharmacological and genetic inhibition of FAK reduces insulin secretion (87) and disrupts secretion targeting *in vitro* (12, 31), while *in vivo* knockout of pancreas-specific FAK results in impaired GSIS and diminished glucose tolerance (88). Although the recruitment and activation of FAK appears essential for normal GSIS, further downstream pathways are currently unclear. Other kinase-associated pathways, such as the extracellular signal-related kinase (ERK), have similarly been shown to regulate GSIS (89), however further investigation into specific signaling cascades will be required to expand on the pathways responsible for modulating β-cell function.

## Involvement of Islet Vascular Cells in Diabetes

Abnormalities in the islet vasculature may drive β-cell dysfunction and diabetes progression. Changes in pericyte function and mass has been implicated in obesity and diabetes (6, 7, 26, 90). Pancreatic pericytes were recently demonstrated to express the diabetes gene transcription factor 7-like 2 (TCF7l2) (26). Polymorphism in TCF7L2 (TCF4) strongly correlates with an increased risk of type 2 diabetes (91). Pericyte-specific inactivation of Tcf7l2 impairs glucose homeostasis due to aberrant insulin production and GSIS (26). This impairment has been associated with reduced expression levels of genes associated with β-cell function and maturity, including *MafA*, *Pdx1* and *NeuroD1*. Furthermore, pancreatic pericytes are shown to produce secreted factors in a Tcf7l2-dependent manner that potentially support β-cell function and glucose response (26). Diabetic retinopathy is characterized by an early loss of retinal pericytes under hyperglycemic conditions (92, 93). Loss of pericytes in the liver and brain leads to endothelial hyperplasia and abnormal vascular (94, 95). In the islets, progression of type 2 diabetes is associated with and may be contributed to by a gradual loss of pericytic coverage of islet capillaries (96).

As β-cell function declines and diabetes progresses, poorly controlled blood glucose levels in the form of chronic hyperglycaemia contributes significantly to abnormal protein glycation throughout the islets and other non-pancreatic tissues (97, 98). The advanced glycation end-products (AGEs) formed by this process are implicated in both worsening β-cell function as well as in development of long-term diabetic complications including diabetic retinopathy (99, 100), nephropathy (100, 101), and decreased insulin sensitivity in adipose tissues (102). Furthermore, islets/β-cells exposed to AGEs in culture shown to have impaired GSIS and other functional defects (103, 104). Although not currently clear, effects on β-cell endocrine function may, in part, be mediated by effects on the ECM proteins of the basement membrane, which are generally long-lived proteins and therefore more susceptible to accumulating effects of glycation.

Both type 1 and type 2 diabetes have been associated ECM abnormalities: progression of type 1 diabetes-related β-cell destruction is correlated with the amount of leukocyte-induced damage to the peri-islet basement membrane (105), while type 2 diabetes islets exhibit thicker, less branched intra-islet capillaries (106) with increased fibrosis surrounding the vasculature (107). Furthermore, pancreatic pericytes can convert to myofibroblasts (96) which leads to aberrant ECM production and tissue fibrosis and would further contribute to impaired β-cell function. Specifically for AGE-related changes to ECM structure, AGE increases crosslinking of the ECM to increase stiffness (108) which may impact on the local islet environment and may inhibit cellular signaling and behaviour. Alteration in ECM stiffness is associated with dysfunction in numerous well-studied disease states, including cancer (109–111), cardiovascular disease (112), and other fibrotic diseases (113, 114). Additionally, the receptor for AGEs (RAGE) is expressed by both endothelial cells and pericytes (115). Along with AGE-triggered basement membrane modification (116), AGE receptors are thought to be involved in the triggering of retinal pericyte apoptosis that occurs in diabetic retinopathy (115) - it may be that islet pericytes undergo similar apoptotic signaling, further impacting pancreatic endocrine function.

## Concluding Remarks

The islet vasculature affects various aspects of pancreatic function and GSIS through both blood flow-dependent and -independent pathways as summarized in **Table 1**. While each of the vascular components, namely endothelial cell and pericytes, are known to individually support β-cell function, whether these cells have a synergistic effect are yet to be directly studied. For example, heterotypic interactions of pericytes and endothelial cells are required for vascular basement membrane assembly in many tissues but this has not yet been shown in the pancreas. Further, whether direct interactions between islet endothelial cells and pericytes affect the other’s production and secretion of factors, thus influencing their ability to support β-cells, is yet to be uncovered.

**Table 1:** Islet Vascular Cell Features (Summary Table).

Feature	Endothelial cells	Pericytes
Cell markers	CD31 (117, 118) CD146, CD105 (118) nephrin (119) von Willebrand’s factor (118)	NG2, PDGFR β (120)
Known effects on β -cells	Gene expression (25) Insulin secretion (7) Replication (5, 25)	Gene expression (26, 27, 77) GSIS (23, 26, 27) Replication (22) Regulation of islet blood flow (21)
Factors secreted	Connective tissue growth factor (CTGF) (53) Thrombospondin (TSP)-1 (60)	Nerve growth factor (NGF) (23) Bone morphogenetic protein 4 (BMP4) (26)
Basement membrane components produced	Collagen IV Laminin α4 Laminin α5 (25)	Collagen IV Laminin α2 Laminin α4 Nidogens Perlecans (77)
Immunoregulatory function	Recruit pancreatic macrophages (5)	Unknown in pancreas Leukocyte trafficking/activation in brain and kidney (32–34) Production of anti-inflammatory factors (33, 121, 122)
Implications in diabetes	Activation of AGE receptors may contribute to progressive complications (115) Alterations of vasculature in diabetic islets (107) and secreted basement membrane (106) associated with disease-related islet damage (105) and malfunction (107)	Type 2 diabetes is associated with a reduced density (21) Support of β-cells depends on the diabetes gene TCF7L2 (26) Transform to myofibroblast during stress (96)

## Author Contributions

GB, CB, PT, and LL wrote and edited the manuscript. All authors contributed to the article and approved the submitted version.

## Funding

Project funding was obtained from the National Health and Medical Research Council (APP1128273 and APP1146788; to PT), the Israel Science Foundation (ISF; Grant agreement no. 1605/18; to LL), and the European Union’s Horizon 2020 Research and Innovation Programme (Grant agreement no. 800981; to LL).

## Conflict of Interest

The authors declare that the research was conducted in the absence of any commercial or financial relationships that could be construed as a potential conflict of interest.
